# Unpuzzling Friunavirus-Host Interactions One Piece at a Time: Phage Recognizes *Acinetobacter pittii* via a New K38 Capsule Depolymerase

**DOI:** 10.3390/antibiotics10111304

**Published:** 2021-10-26

**Authors:** Rita Domingues, Ana Barbosa, Sílvio B. Santos, Diana Priscila Pires, Jonathan Save, Grégory Resch, Joana Azeredo, Hugo Oliveira

**Affiliations:** 1 Centre of Biological Engineering, University of Minho, 4710-057 Braga, Portugal; ritadomingues1898@gmail.com (R.D.); pg38833@alunos.uminho.pt (A.B.); silviosantos@ceb.uminho.pt (S.B.S.); priscilapires@deb.uminho.pt (D.P.P.); 2 Department of Intensive Care Medicine, Inselspital, Bern University Hospital, University of Bern, CH-3010 Bern, Switzerland; savejonathan@gmail.com; 3 Centre for Research and Innovation in Clinical Pharmaceutical Sciences, Lausanne University Hospital, CH-1011 Lausanne, Switzerland; gregory.resch@chuv.ch

**Keywords:** *Acinetobacter baumannii*, bacteriophage, tailspike, depolymerase, anti-virulence, capsule

## Abstract

*Acinetobacter pittii* is a species that belong to the *Acinetobacter calcoaceticus-baumannii* complex, increasingly recognized as major nosocomial bacterial pathogens, often associated with multiple drug-resistances. The capsule surrounding the bacteria represents a main virulence factor, helping cells avoid phage predation and host immunity. Accordingly, a better understanding of the phage infection mechanisms is required to efficiently develop phage therapy against *Acinetobacter* of different capsular types. Here, we report the isolation of the novel *A. pittii*-infecting Fri1-like phage vB_Api_3043-K38 (3043-K38) of the *Podoviridae* morphotype, from sewage samples. Its 41,580 bp linear double-stranded DNA genome harbours 53 open reading frames and 302 bp of terminal repeats. We show that all studied *Acinetobacter* Fri1-like viruses have highly similar genomes, which differentiate only at the genes coding for tailspike, likely to adapt to different host receptors. The isolated phage 3043-K38 specifically recognizes an untapped *Acinetobacter* K38 capsule type via a novel tailspike with K38 depolymerase activity. The recombinant K38 depolymerase region of the tailspike (center-end region) forms a thermostable trimer, and quickly degrades capsules. When the K38 depolymerase is applied to the cells, it makes them resistant to phage predation. Interestingly, while K38 depolymerase treatments do not synergize with antibiotics, it makes bacterial cells highly susceptible to the host serum complement. In summary, we characterized a novel phage-encoded K38 depolymerase, which not only advances our understanding of phage-host interactions, but could also be further explored as a new antibacterial agent against drug-resistant *Acinetobacter*.

## 1. Introduction

*Acinetobacter calcoaceticus-baumannii* complex (ACB complex) is a leading opportunistic nosocomial pathogen. This complex consists of five closely related species: *Acinetobacter baumannii*, *Acinetobacter nosocomialis*, *Acinetobacter pittii*, *Acinetobacter seifertii* and *Acinetobacter dijkshoorniae*, and also one non-pathogenic species: *Acinetobacter calcoaceticus*. Treatment of ACB complex species associated infections is extremely difficult due to their multi-resistance to several classes of antibiotics [1]. The alarming emergency of carbapenem-resistant clones, together with the lack of viable antimicrobial options, has placed *A. baumannii* as the leading priority pathogen since 2017 by the World Health Organization. However, this should be carefully interpreted, as hospital routines cannot easily distinguish among species of the ACB complex, often identifying *A. baumannii* by default. Accordingly, recent studies have reported that *A. pittii* is sometimes more prevalent than *A. baumannii* in blood cultures [2].

A thick capsular polysaccharide coat typically displayed in ACB complex strains is considered a major virulence factor [3]. Different genes responsible for the synthesis, assembly and exportation of capsule polysaccharides (K types) have evolved to produce variable serotypes, as a consequence of constant interactions of the bacterium with the host immunity, other eukaryotic cells and bacteriophage (phages) [4]. Currently, ACB complex is known to display more than 125 different K types [5]. This variety might also be related to different degrees of clinical manifestation of infections and antibiotic resistances.

ACB complex phages (i.e., viruses infecting specifically ACB bacteria) have been investigated as a possible alternative medicine to antibiotics, in the frame of the so-called phage therapy. In 2017, a total of 42 *Acinetobacter* phage genome sequences were available in public databases. Comparative analysis of their genomes and proteomes revealed that *Acinetobacter* phage population was composed of six groups. Phages of the subfamily *Autographivirinae* and *Fri1virus* genus at the time were represented by nine phages of *Podoviridae* morphotype (AB3, Abp1, IME-200, phiAB1, phiAB6, PD-AB9, PD-6A3, WCHABP5). All have evolved to encode tailspikes with capsular depolymerase activities that specifically recognize bacterial K types, allowing phages to infect the bacterium. Since then, *Acinetobacter* phage genome numbers have increased by three-fold. Although *Acinetobacter* phages with capsular depolymerases have been found in different taxonomic groups, they were predominantly identified in *Fri1virus*-like phages. Currently, 17 distinct ACB complex K-specific depolymerases have been identified in phage proteomes (K1–2, K9, K19, K27, K30, K32, K37, K44–45, K47–48, K87, K89, K91, K93 and K116) [6,7,8,9,10].

In the continuous search to disclose the full diversity of phage-host capsular interactions, we isolated a novel phage against an untapped *A. pittii* K38 capsule type. The Fri1-like phage vB_Api_3043-K38 (3043-K38) encodes a novel tailspike protein responsible for its specific interaction with *A. pittii* K38. We report here the isolation and full characterization of this new tailspike protein carrying a K38-depolymerase activity, thus advancing our current knowledge about the mechanisms of phage interaction with bacterial strains of the ACB complex.

## 2. Results

### 2.1. A. pittii Ap45 Has a OXA β-Lactamase and a K38 Capsular Type

Ap45 strain was isolated from a wound of a patient at the University Hospital of Lausanne (CHUV), Switzerland. Sequencing yielded 3,491,380 million 150-bp paired-end reads, representing an average coverage of 65.6x. *De novo* assembly of the Ap45 genome yielded 39 contigs (>500 bp) with a total size of 4,058,915 Kb, and a GC-content of 38.7%. The assembly N50 value was 295,941 kb. Average nucleotide identity (ANI) against type strains of *A. pittii* (APQP00000000) and *A. baumannii* (CP046654) were 97.44% and 87.53%, respectively. ANI values ≥ 96% strongly indicated identity at the species level [11], i.e., the genome was clearly derived from an *A. pittii* strain. The genome encoded 3,918 genes, 62 tRNA genes, 4 ncRNA, 3 rRNA genes, and 1 tmRNA. Assembled contigs were also predicted to encode an OXA-213-like β-lactamase OXA-500 oxacillinase gene (*bla*_OXA-500_). Additionally, a capsular synthesis locus of the K38 type was identified with good confidence (i.e., 91% coverage and 98% identity).

### 2.2. Phage 3043-K38 Is a New Fri1-like Virus Infecting the A. pittii K38 Capsule Type

**Figure 1:**
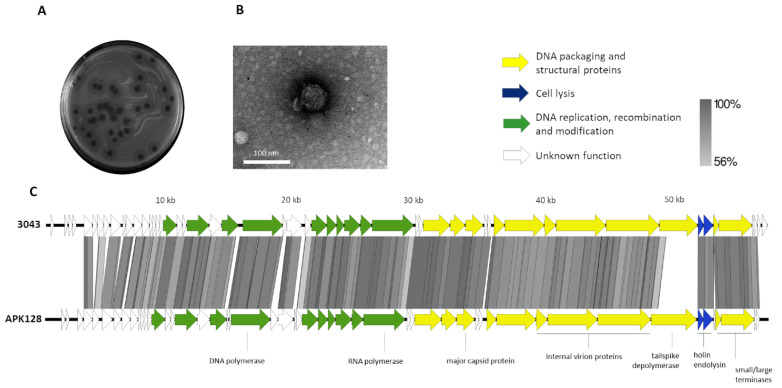
Morphological and genomic analysis of phage 3043-K38. (**A**) morphological images of the phage plaque and (**B**) TEM micrographs (scale bar, 100 nm) negatively stained with 2% uranyl acetate. (**C**) Genomic map of phage 3043-K38 (53 predicted proteins are coloured according to their function) compared with *Acinetobacter* phage APK128 using the Easyfig [12] program and TBLASTX comparisons.

We isolated a new phage named vB_Api_3043-K38 (referred to as 3043-K38) infecting only K38 strains out of 29 different *Acinetobacter* K types tested. After overnight incubation, phage produced clear plaques (~2 mm in diameter) surrounded with halos (~7 mm in diameter) (Figure 1A). While phage plaques maintained their size, the halos increased over time. Based on transmission electron microscopy (TEM) analysis, 3043-K38 harboured an icosahedral symmetric capsid (56 nm vertex to vertex) and a short noncontractile tail (9 nm head edge to tail end), featuring a *Podoviridae* morphotype (Figure 1B).

The phage 3043-K38 had a 41,882-bp double-stranded DNA with 302-bp direct terminal repeats (DTRs), and a GC content of 39.3%. 53 genes were predicted to encode proteins with function related to DNA replication, repair and modification, cell lysis and DNA packaging as well as structural proteins (Figure 1C). Phage 3043-K38 shared substantial nucleotide identity (80%) and (>85%) genes with many *Acinetobacter* Fri1-like viruses, such as vB_AbaP_APK128 (MW459163), vB_AbaP_APK116 (MN807295), and vB_ApiP_P2 (NC_042007). The high genomic and proteomic similarity between our phage 3043-K38 and the above-mentioned viruses that belong to the *Autographiviridae* family and *Friunavirus* genus, support the *Podoviridae* morphotype of the phage 3043-K38. Whole genome comparison showed that *Acinetobacter* Fri1-like viruses have highly similar genomes, and that a main difference can be found at the gene encoding for the tailspike depolymerase (Figure 1C).

### 2.3. Tailspike Carries a Novel K38 Depolymerase Activity

**Figure 2:**
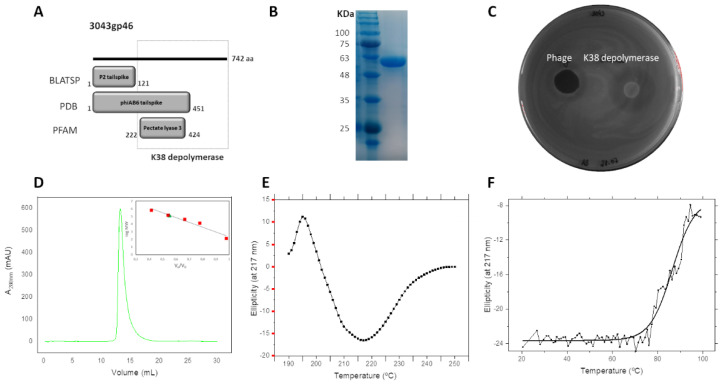
Tailspike structural analysis. (**A**) Bioinformatics analysis of the tailspike gene, with a putative capsular depolymerase region identified. (**B**) 12% SDS-PAGE separation gel (GRS Protein Marker) of the purified protein. (**C**) Spot test of the capsular depolymerase on agar plates against K38 cells. (**D**) size exclusion chromatography of samples loaded to a Superose 6 10/300 GL column (mAu, micro absorbance unit). Capsular depolymerase was eluted as a single peak corresponding to a molecular weight of 146.25 kDa, based on the calibration curve of log MW vs. Ve/V0 (Ve, elution volume; V0, void volume) generated with calibration standards (Merk). (**E**) Circular dichroism analysis of the purified protein (1 mg; measured in the far-UV) and (**F**) its thermal unfolding acquired at 217 nm. Circular dichroism spectroscopy was performed with proteins dialyzed in potassium phosphate buffer (pH 7.0).

Like other described phage tailspikes [13], the 742-aa-long tailspike of phage 3043-K38 is predicted to have a N-terminal anchor domain and a centre depolymerase domain (Figure 2A). A putative C-terminal chaperone described in some homologs was not detected. At the primary sequence level, only the N-terminal domain (1–121 aa) was similar to other *Acinetobacter* phage tailspikes (>70% aa identity). At the structural level, the N-terminal and centre regions (1–451 aa) were similar to the *Acinetobacter* phage phiAB6 tailspike (E-value of 1.6 × 10^−10^). PFAM also predicted a pectate lyase 3 domain in the middle region (201–225 aa, protein family PF12708.6) although with low identity (E-value of 2.1). This region has been associated with beta-helix enzymes with capsular depolymerase activities [6].

We decided to heterologously express the tailspike middle and C-terminal region (147–742 aa) in *E. coli,* predicted to encode the capsular depolymerase domain and its putative chaperone, respectively (Figure 2B). Drop tests showed halos only against the K38 strain, mimicking the haziness of the phage 3043-K38 plaques (Figure 2C). The K38 depolymerase was active in the 0.0001 µM range. Moreover, it was able to degrade extracted exopolysaccharides, from 20 °C to 90 °C (Supplemental Figure S1).

### 2.4. K38 Depolymerase Is a Heat-Resistant Trimeric Protein

The his-tagged, expressed K38 depolymerase corresponds to a 630-aa protein and a heterologously theoretical mass of 67 kDa, which is contrasted with its molecular mass of 55 kDa, obtained after electrophoresis in polyacrylamide gel under denaturing conditions (Figure 2B). In addition, analytical size-exclusion chromatography showed a single homogeneous peak at around 146 kDa (peak at 13.28 mL), close to the trimeric value calculated from the electrophoresis (i.e., 165 kDa). This indicated that the protein likely forms trimers in solution (Figure 2D).

Circular dichroism (CD) was performed to evaluate the K38 depolymerase secondary structure content. CD spectrum demonstrated that the K38 depolymerase is a β-sheet-rich protein, as it displayed a single characteristic minimum peak at 217 nm (Figure 2E). With a complementary analysis of a second PSIPRED program, we confirmed that K38 depolymerase has a higher content of β-structures (43%), followed by α-helices (18%), being the remaining regions coiled. The CD was also used to analyse the structure thermostability of the K38 depolymerase. Thermal denaturation produced a sigmoidal curve, reflecting protein unfolding (Figure 2F). The melting temperature (T_m_) i.e., midpoint, was measured at 85.6 °C.

### 2.5. Recombinant K38 Depolymerase Quickly Removes Capsules, Making Bacterial Cells Immune to Phage

**Figure 3:**
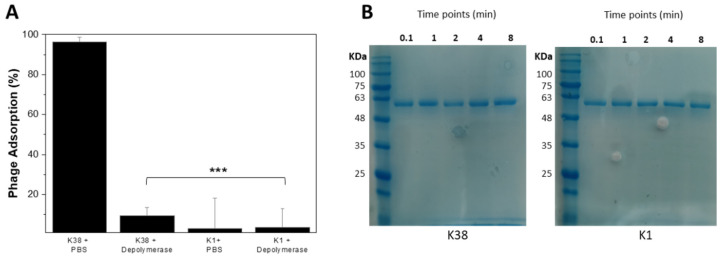
Tailspike interaction with capsules. (**A**) Adsorption rate of phage 3043-K38 particles in K38 host cells pre-treated with PBS (control) or K38 depolymerase at 1 µM (test), obtained by subtracting the titre before (total phage titre) and after centrifugation (non-adsorbed phage titre). Error bars represent standard deviation for three repeated experiments. Statistical significance was determined by Student’s *t*-test (***, *p* < 0.001). (**B**) Binding of the K38 depolymerase to K38 cells assayed as described in the material and methods. In brief, recombinant K38 depolymerase was incubated with K38 (sensitive to the enzyme) or K1 cells (not sensitive to the enzyme) at different time points (0.1, 1, 2, 4, 8 min). Immediately afterward, samples were centrifuged, and the supernatant was collected and loaded in a 12% SDS-PAGE gel.

To deepen our understanding of the capsular depolymerase role during phage infection, we performed two independent experiments. In the adsorption assay (Figure 3A), phage 3043-K38 adsorbed with high efficiency (96.3% adsorption) on its host K38 but adsorption was significantly affected on depolymerase-treated cells (6.3% adsorption) (*p* < 0.001). In contrast, the adsorption of 3043-K38 was observed on neither non-treated nor on depolymerase-treated K1 cells (i.e., not susceptible to 3043-K38 or the recombinant depolymerase, 2.9% and 3.6% adsorption, respectively). Binding assays showed that the interaction between the recombinant K38 depolymerase and the capsule is transient and fast, the enzyme being released already by 10 s (Figure 3B).

### 2.6. K38 Depolymerase Does Not Synergise with Antibiotics

With the goal of understanding if the capsule disruption could increase susceptibility to antibiotics, preliminary experiments were performed to analyse whether the K38 depolymerase synergized with antibiotic treatments in biofilms. The three antibiotics with lowest MIC (Supplemental Figure S2) were selected for these assays: ciprofloxacin (MIC = 1 µg/mL), tetracycline (MIC = 1 µg/mL) and gentamicin (MIC = 2 µg/mL). We performed co-treatments using antibiotics (5xMIC) in combination with the K38 depolymerase (1 µM) against 24 h preformed biofilms in 96-well microtiter plates. Ciprofloxacin, tetracycline and gentamicin treatments reduced approximately 2, 1 and l logs, compared with the control group and respectively (Supplemental Figure S3). However, no differences were observed between the use of antibiotics alone and the combined treatments (antibiotic + K38 depolymerase) in all tested conditions.

### 2.7. K38 Depolymerase Increased Susceptibility of K38 Cells to Host Immunity

**Figure 4:**
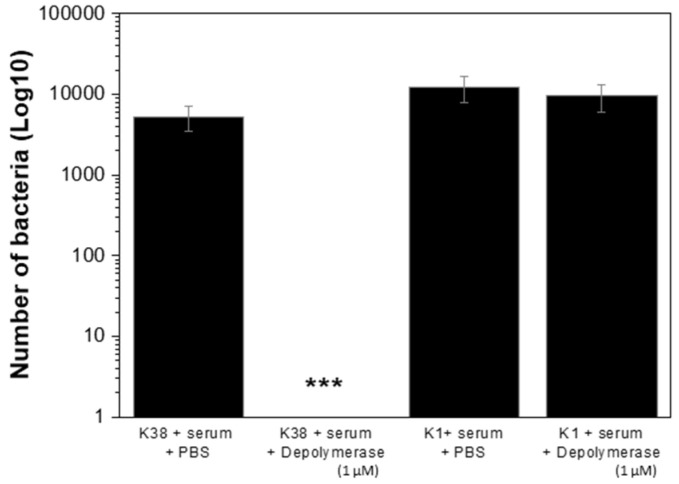
Serum assay. The survival of phage host K38 and non-host K1 strains against the serum complement collected from healthy human subjects, was assessed in the presence of PBS (control) or K38 depolymerase at 1 µM (test). Significance was determined by Student’s *t* test (***, *p* < 0.001).

The capsular degrading activity was also assessed as a potential anti-virulence strategy against *A. pittii*. Interestingly, while non-treated K38 cells were fully resistant to serum, addition of purified recombinant K38 depolymerase to K38 cells led to full susceptibility to serum (Figure 4). Indeed, in the presence of the depolymerase, the starting 4-log bacterial inoculum was reduced below detection limit (<10 CFU/mL). As expected, K38 depolymerase had no effect on the resistance of K1 cells to the serum (Figure 4). In all cases, heat-inactivated serum combined with the K38 depolymerase had no antibacterial effect (data not shown). These experiments suggested a specific anti-virulence effect of the depolymerase towards K38 cells in the presence of the host complement system.

## 3. Discussion

A first step in the interaction between phages and their bacterial prey involves the binding of the phage to bacterial surface receptors through receptor binding proteins usually carried on the phage tail fibers and/or tailspikes. One of the main driving forces in the evolution of phage genomes is the acquisition of novel receptor binding proteins to adapt to new host receptors, in the endless “arms race” between host defences and phage counter defences.

**Figure 5:**
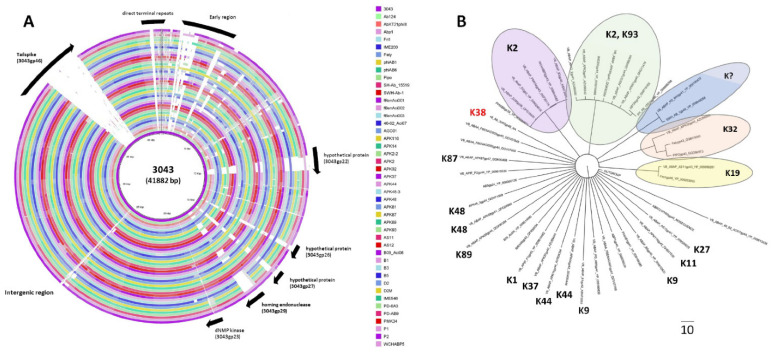
Horizontal movement of Acinetobacter fri1-like phage tailspikes. (**A**) Comparative genomics maps of all 45 fri1-like viruses deposited in GenBank (June 2021), plus the newly isolated phage 3043-K38 and (**B**) phylogenetic relationships of their predicted capsular depolymerase (pectate lyase 3) domains located at the tailspikes, using Muscle multiple sequence alignments and observed by the neighbor-joining analysis (using a branch-and-bound algorithm with 100 bootstrap replicates), and using the E. coli phage K1F capsule depolymerase (YP_338127) as an outgroup. Colors identify clusters of depolymerase sequences predicted to target similar capsule types.

Previously, we studied the interaction of Fri1-like ACB complex phages of the *Autographiviridae* family. By isolating 12 new phages, we demonstrated that their genomes were highly similar but encoded specific capsular depolymerase domains to specifically recognise different host capsules [6]. Later, Fri1-like phages have been reported to interact in total with 17 distinct *Acinetobacter* capsules (K1–2, K9, K19, K27, K30, K32, K37, K44–45, K47–48, K87, K89, K91, K93 and K116) [6,7,8,9,10]. Comparing the genome of phage 3043-K38 targeting an untapped K38 capsule with the 45 ACB complex Fri1-like phages deposited at GenBank, we confirmed our previous observations that amongst highly similar genomes, high variability could be found mainly between genes encoding for the tailspike (Figure 5A). Their genomes were highly similar, presenting only some synteny breaks in some hypothetical genes, homing endonuclease, a dNMP kinase, but mostly at the tailspike genes. If fact, only the center-end of the tailspikes coding for the capsular depolymerase activity varyied, which can be interpreted as a specific genomic-switch to quickly adapt phages to new hosts. This impressive genomic similarity, albeit the distinct geographical and environmental isolations sources, is an obvious statement of the strong selection to maintain the same genetic structure of all Fri1-like ACB complex phages. Our hypothesis is that these small viruses (typically 40 Kb), need specialized enzymes (depolymerases) to reach the inner surface receptors and initiate the infection their otherwise too-small tails would not reach.

Combining the experimental data of reported *Acinetobacter* Fri1-like tailspikes with depolymerase activities [6,7,8,9,10] and multiple sequence alignments of their domains, one can appreciate the vast diversity of capsular depolymerases that interact with *Acinetobacter* hosts, which is known to have 125 different capsule types (Figure 5B). Sequences grouped (cluster) by similarity are expected to behave similarly, while single sequences (like the K38 depolymerase) are unique (Figure 5B). Our analysis predicts that from the 46 Fri1-like ACB complex phages analysed, 42 are capsule depolymerases (pectate_lyase_3, PF12708 domains), 2 are esterase depolymerase that modify, but not degrade the LPS (esterases with Lipase_GDSL PF00657 domains) and 2 have unique domains (AbKT21phiIII, vB_AbaP_46-62_Aci07). Owing to the horizontal movement of the tailspikes in Fri1-like ACB complex phages genomes observed, it is likely that these unique domains behave also as depolymerases.

Fri1-like ACB complex phages encode tailspike with capsular depolymerases that assemble as trimers. The variation between the theoretical trimeric molecular mass with the value obtained in gel filtration experiments, agrees with previous observations reporting that the C-terminal domain functions as a chaperone to help the protein to oligomerise into trimers, and is then autoproteolytically cleaved [14,15]. The K38 depolymerase study presented here suggests that the interaction with the capsule may be faster (in the second range) when compared with other lipopolysaccharide depolymerases described [16]. Without surprise, the K38 depolymerase harbours a rich β-structure that enables the enzyme to endure high thermal stresses, with T_m_ > 65 °C as observed previously with other *A. baumannii*, *K. pneumoniae* and *E. coli* phage capsular depolymerases [7,17,18].

Potential biotechnological applications of the recombinant depolymerase were also explored, here. We showed that the recombinant K38 depolymerase degrades capsules, making cells immune to phage predation. This shows that a combinatorial use of the phage and its recombinant depolymerase is not advisable for treatment of bacterial infections. By stripping cells from the capsule coat, K38 depolymerase also increased susceptibility of bacterial cells to the complement. This agrees with recent findings from similar enzymes, the *Acinetobacter* phage B9 and B3 capsular depolymerases [7,19,20]. However, K38 depolymerase had no additive or synergistic effect with antibiotics to control biofilms. To the best of our knowledge, only a single recombinant depolymerase derived from *Aeromonas punctata* was reported to synergize with gentamicin against a *K. pneumoniae* biofilm, but its origin and polysaccharide substrate remains to be fully elucidated [21]. While a synergistic depolymerase-antibiotic combination may be depended of the antibiotic mechanism of action, the ability to control biofilms may be ultimately related with the prevalence of capsule polysaccharides in the extracellular matrix, which will be strain-dependent.

In summary, by isolating a novel K38 depolymerase we contributed to unpuzzling Friunavirus-host interactions and highlighted a potential application of these enzymes to control bacterial infections. Due to the high specificity of these capsular depolymerases for a given capsule type, epidemiologic studies need to be first conducted in order to understand the most prevalent ACB complex capsular types circulating in human clinical isolates. Only then, the anti-virulence therapy based on capsular depolymerases could be directed and potentially become an alternative to current antimicrobials options, which are becoming less effective against, drug-resistant infections, such as the ones caused by ACB species.

## 4. Material and Methods

### 4.1. Bacterial Strains and Culture Conditions

*A. pittii* Ap45 strain was isolated from the drainage liquid collected from a wound of a patient at the University Hospital of Lausanne (CHUV), Switzerland. Other considered *Acinetobacter* strains belonged to the collections of Alexandr Nemec (NIPH and ANC strains) and the Institute Pasteur (CIP strains), covering a range of 29 different *Acinetobacter* K types (K1–3, K7, K9, K11, K14–15, K19, K22, K30, K32–33, K35, K37–38, K40, K43–49, K57, K73, K83–85) [6,7,19]. Unless otherwise stated, all strains were grown at 37 °C in Tryptic Soy Broth (TSB) or on Tryptic Soy Agar (TSA, 1.2% (wt/vol) agar).

### 4.2. Bacterial Sequencing and Annotation

*A. pittii* Ap45 genomic DNA was isolated using the Qiagen DNeasy Blood and Tissue Kit following the manufacturer’s recommendations. Next, bacterial genomic library preparation was performed by Eurofins Genomics Germany GmbH (Ebersberg, Germany) with an optimized protocol and standard Illumina adapters. Sequencing was performed at Eurofins Genomics Germany GmbH (Ebersberg, Germany) with Illumina technology, NovaSeq 6000 (read mode 2 × 150 bp). Sequencing data were assembled with Geneious Prime 2020 (Biomatters Ltd., Auckland, New Zealand). Contigs shorter than 500 bp or with less than eight-fold coverage were removed. Assembled contigs were annotated using the NCBI Prokaryotic Genome Annotation Pipe-line v4.12 [22]. JSpeciesWS was used to compute pairwise average nucleotide identity (ANI) values among genomes [23]. Acquired antibiotic resistance genes were screened using ResFinder 4.0 [24]. Kaptive was used to identify the *Acinetobacter* K locus for capsular polysaccharide types [25].

### 4.3. Phage Isolation and Propagation

Phage 3043-K38 was isolated using the procedures previously described [26]. Briefly, phages specific for *A. pittii* Ap45 were amplified from raw sewage water samples (Vidy wastewater treatment plant, Lausanne, Switzerland) supplemented with Luria Broth medium and the *A. pittii* Ap45 strain. After incubation for 24 h at 37 °C and 200 rpm, samples were centrifuged (4000× *g*, 15 min) and the supernatant filtered (0.45-μm syringe filters). Plaque-forming units (PFU) were obtained using double-layer assays with serial dilutions of the filtered supernatant. Individual PFU were spread into new bacterial lawns at least three times to ensure selection of a single phage, which was named vB_Api_3043-K38 (3043-K38).

*A. pittii* phage 3043-K38 was propagated on *A. pittii* Ap45 strain (K38 capsular type). Briefly, mid-exponential cultures (OD_620nm_ of 0.3) of *A. pittii* Ap45 grown in TSB were infected with the 3043-K38 at a multiplicity of infection (MOI) of 0.01 at 37 °C for 4 h. The lysate was centrifuged (9000× *g*, 10 min) and the supernatant was filtered at 0.22 µm and titrated through double-layer agar assay, following standard procedures [27].

### 4.4. Electron Microscopy

The phage was imaged in a Jeol JEM 1400 transmission electron microscope (TEM), precisely as previously described [28].

### 4.5. Phage Genome Sequencing and Annotation

Genomic DNA was extracted and purified using the phenol-chloroform extraction method, as described elsewhere [27]. Whole genome library was generated by TruSeq^®^ Nano DNA Library Prep Kit, sequenced in lllumina MiSeq with a 300 bp paired-end sequencing read configuration (Stabvida, Portugal) and, de novo, assembled in Geneious Prime. The assembled genome was annotated with MyRAST [29], BLAST [28], tRNAscan-SE v2.0 [30] and HHpred [31] with default parameters.

### 4.6. Tailspike Cloning, Expression and Production

The gene coding for a deletion mutant lacking the N-terminal domain (i.e., amino acid residue 147–742) of the tailspike encoded by *gp46*, also referred to as capsular depolymerase, was amplified with phusion polymerase (Thermo Scientific, Waltham, MA, USA) from the phage genomic DNA using the following primers: Fw 5′-GGATCCCAGGAAGTACGTTCGGC-3′ (with BamHI restriction site); Rv 5′-CTCGAGTTAACTCGGTGTAAGTGTAGTACC-3′ (with XhoI restriction site). The gene was cloned in pET28a (Addgene) and transferred into electrocompetent *Escherichia coli* BL21 cells for recombinant expression. Protein synthesis was induced in mid-exponential cultures (OD_620nm_ of 0.5) with 1 µM of isopropyl-β-D-thiogalactoside (IPTG) at 21 °C under agitation for 16 h. Next, cells were pelleted (9000× *g*, 30 min), resuspended in lysis buffer (50 mM Tris-HCl pH 8.0, 300 mM NaCl), and mechanically disrupted by three freeze/thaw cycles and sonication (Cole-Parmer, Ultra-sonic Processors) for 8–10 cycles (30 s pulse, 30 s pause, 40%). Capsular depolymerase was purified through immobilized metal affinity chromatography (IMAC) on nickel columns using an imidazole gradient (25 to 250 mM). Eluted samples were separated on 12% polyacrylamide gel electrophoresis (SDS-PAGE) and dialyzed in 10-mM phosphate buffered saline (PBS, pH 7.5). Next, protein samples were further purified by size exclusion chromatography performed on an Azura FPLC system (KNAUER) at 4 °C. Sample was applied to a Superose 6 10/300 GL column (GE Healthcare, Chicago, IL, USA) in 10-mM PBS (pH 7.5). Elution was conducted at a flow rate of 0.75 mL/min and the protein was detected at A_280_. Molecular weight was estimated using protein standards (Merck). The purified protein was quantified by the Pierce™ BCA Protein Assay Kit (Thermo Scientific, Waltham, MA, USA).

### 4.7. Phage Host Range and Depolymerase Activity Spectrum

A panel of 29 different *Acinetobacter* K types (K1–3, K7, K9, K11, K14–15, K19, K22, K30, K32–33, K35, K37–38, K40, K43–49, K57, K73, K83–85) [6,7,19] was used to check 3043-K38 host range and the activity spectrum of the recombinant capsular depolymerase. After overlaying overnight cultures in Tryptic Soy Agar (TSA) plates, 5-µL drops of phage 3043-K38 (10^8^ PFU/mL) or of the recombinant capsular depolymerase (1 µM) were spotted and plates incubated at 37 °C overnight. Susceptibility to phage or depolymerase was defined as appearance of clear or opaque lysis zones (halos) at the deposition sites, respectively.

### 4.8. Circular Dichroism Spectroscopy

The secondary structure of the capsular depolymerase (10 µM) was analysed by circular dichroism (Jasco J-1500 spectrometer) measuring the ellipticity spectrum (average of three accumulations with baseline correction) from 190 to 250 nm, with 1 nm steps, scanning speed of 20 nm/min, high sensitivity and 16-s response time. Additionally, thermal unfolding was analysed by monitoring the ellipticity (at 217 nm), heating the protein (1 °C/min) from 25 °C to 100 °C. Melting curves were obtained after fitting the data in a Boltzmann sigmoidal function.

### 4.9. Binding Assay

This assay was performed as previously described, with minor modifications [16]. Overnight cultures of K38 strain (3043-K38 host) and K1 strain (3043-K38 non-host) were adjusted to an optical density (OD_600nm_) of 1.0. A small fraction of the cultures (6 mL) was pelleted (5000× *g*, 5 min at 4 °C) and resuspended in 200 μL of 10-mM PBS (pH 7.5) containing the tailspike (2 µM final concentration). The mixture was incubated at 37 °C with agitation. At different time points, the cells were pelleted (20,000× *g,* 30 s at room temperature) and the supernatant (30 μL) was recovered and loaded into 12% SDS-PAGE gel stained with Coomassie Blue.

### 4.10. Adsorption Assay

Pull-down assays were performed as previously described, with minor modifications [19]. Overnight cultures of K38 strain (3043-K38 host) and K1 strain (3043-K38 non-host) were diluted to 10^8^ CFU/mL and incubated with PBS or the capsular depolymerase (1 µM final concentration) for 2 h at room temperature. Next, cells were pelleted (8000× *g*, 2 min), washed twice in PBS and incubated with the phage 3043-K38 (MOI of 0.001) at 37 °C for 5 min with agitation. Phage was tittered before (total phage titre) and after centrifugation (non-adsorbed phage titre) in K38 strain. The phage adsorption was obtained by subtracting both and expressing the results in percentage. Averages and standard deviations of three repeated experiments are given. Significance was determined by Student’s *t*-test (***, *p* < 0.001).

### 4.11. MIC Determination and Antibiofilm Assays

The minimum inhibitory concentration (MIC) values of eight different antibiotics (ciprofloxacin, erythromycin, tetracycline, spectinomycin, kanamycin, ampicillin, ceftazidime and gentamicin) were determined for *A. pittii* Ap45 strain in TSB using the broth microdilution method according to a previously described protocol [32]. These assays were performed in triplicate.

Three antibiotics (ciprofloxacin, tetracycline and gentamicin) were selected to perform antibiofilm assays in combination with the K38 depolymerase. For this, overnight cultures of *A. pittii* Ap45 were diluted 1:100 in TSB and 200 µL of the bacterial suspension were added to the wells of 96-well polystyrene microtiter plates (Orange Scientific). The plates were then incubated in an orbital incubator for 24 h at 37 °C and 120 rpm to allow biofilm growth. After biofilm formation, the media and planktonic bacteria were removed, the wells were washed with fresh TSB and incubated with (1) antibiotics (5xMIC), (2) capsular depolymerase (1 µM), (3) antibiotic (5xMIC) + depolymerase (1 µM) or (4) PBS buffer (control). The plates were incubated at 37 °C with agitation (120 rpm) and the colony forming units (CFU)/mL were counted at 6 h and 24 h. Three independent assays were performed.

### 4.12. Human Serum Assay

The anti-virulence properties of the capsular depolymerase were tested in serum as previously described [7]. In brief, overnight cultures of K38 strain (3043-K38 host) and K1 strain (3043-K38 non-host) diluted in TSB to 10^4^ CFU/mL were added to human serum collected from healthy volunteers at a 1:3 volume ratio. Next, infected serum was incubated either with PBS or the capsular depolymerase (1 µM final concentration) for 1 h at 37 °C. Similar samples supplemented with decomplemented serum (at 56 °C for 30 min) were used as controls. Survival bacterial cells were determined by CFUs counts. Averages and standard deviations of three repeated experiments are given. Significance was determined by Student’s *t*-test (***, *p* < 0.001).

### 4.13. Nucleotide Sequence Accession Numbers

*A. pittii* phage vB_Api_3043-K38 genome was deposited in GenBank under accession number MZ593174. The sequencing reads and assembled contigs of the *A. pittii* Ap45 strain are provided under the BioProject accession number PRJNA750264.

## Supplementary Materials

The following are available online at https://www.mdpi.com/article/10.3390/antibiotics10111304/s1. Figure S1. K38 depolymerase activity. Figure S2. MIC values. Figure S3. Biofilm assays.
