# Supplementary material for: Unpuzzling Friunavirus-Host Interactions One Piece at a Time: Phage Recognizes Acinetobacter pittii via a New K38 Capsule Depolymerase
Source: Antibiotics (Basel). 2021 Oct 26;10(11):1304. doi: 10.3390/antibiotics10111304 (PMC8614642; doi:10.3390/antibiotics10111304)
Supplement: Supplementary file 1 [file antibiotics-10-01304-s001.zip › new Supplemental figure S1.pdf]

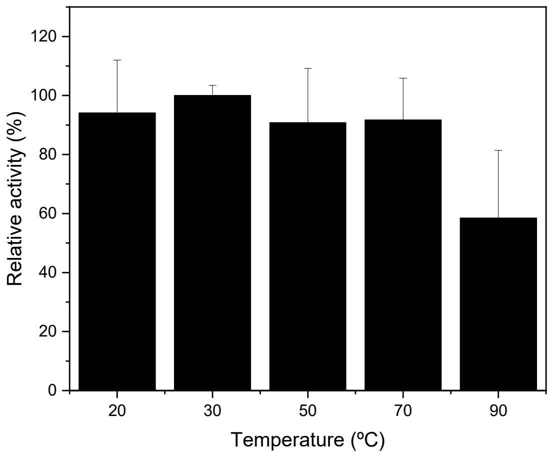

Supplemental Figure S1. K38 depolymerase activity. The enzyme (at 1 µM) was incubated with extracted exopolysaccharides from *A. pittii* Ap45 cells at different temperatures. The results are expressed as relative activity, comparing with the best activity value obtained, 37 °C.
