# Supplementary material for: Unpuzzling Friunavirus-Host Interactions One Piece at a Time: Phage Recognizes Acinetobacter pittii via a New K38 Capsule Depolymerase
Source: Antibiotics (Basel). 2021 Oct 26;10(11):1304. doi: 10.3390/antibiotics10111304 (PMC8614642; doi:10.3390/antibiotics10111304)
Supplement: Supplementary file 1 [file antibiotics-10-01304-s001.zip › new Supplemental figure S3.pdf]

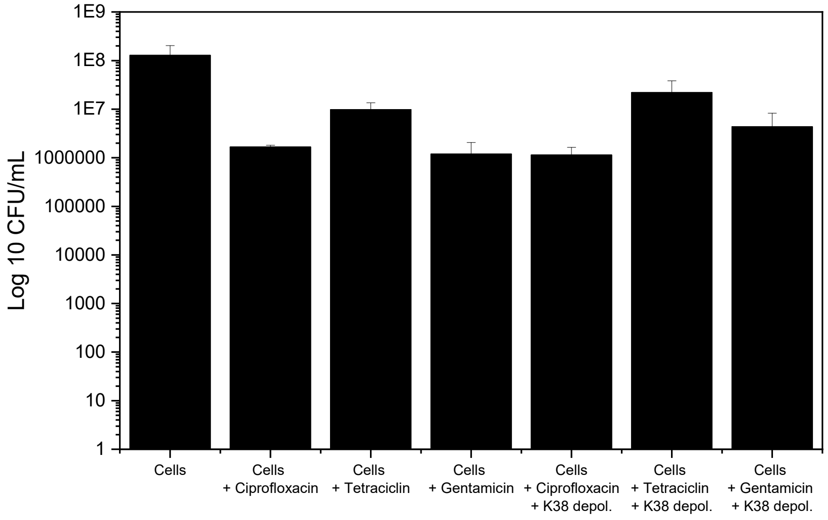

Supplemental Figure S3. Biofilm assays. Preformed biofilms made with *A. pittii* Ap45 cells in 96-well polystyrene microtiter plates were challenged with 1) PBS buffer (control), 2) antibiotics (5xMIC), 3) antibiotic (5xMIC) + depolymerase (1 µM) or 4) capsular depolymerase (1 µM) alone. Colony forming units (CFU)/ml were counted after 24 h.
