# Supplementary material for: Unpuzzling Friunavirus-Host Interactions One Piece at a Time: Phage Recognizes Acinetobacter pittii via a New K38 Capsule Depolymerase
Source: Antibiotics (Basel). 2021 Oct 26;10(11):1304. doi: 10.3390/antibiotics10111304 (PMC8614642; doi:10.3390/antibiotics10111304)
Supplement: Supplementary file 1 [file antibiotics-10-01304-s001.zip › Supplemental figure S2.pdf]

Antibiotic	MIC (µg/ml)	Mechanism of action
Ciprofloxacin	1	Inhibition of DNA replication
Erythromycin	8	Inhibition of protein synthesis
Tetracycline	1	Inhibition of protein synthesis
Spectinomycin	32	Inhibition of protein synthesis
Kanamycin	4	Inhibition of protein synthesis
Ampicillin	128	Inhibition of cell wall synthesis
Ceftazidime	8	Inhibition of cell wall synthesis
Gentamicin	2	Inhibition of protein synthesis

Supplemental Figure S2. MIC values. MICs of 8 different drugs in *A. pittii* Ap45.
